# Inflammation—Insulin Resistance Crosstalk and the Central Role of Myokines

**DOI:** 10.3390/ijms27010060

**Published:** 2025-12-20

**Authors:** Maria-Zinaida Dobre, Bogdana Virgolici, Daciana Costina Andrada Dunca-Stefan, Ioana-Cristina Doicin, Iulia-Ioana Stanescu-Spinu

**Affiliations:** 1 Department of Biochemistry, Faculty of Medicine, Carol Davila University of Medicine and Pharmacy, 050474 Bucharest, Romania; maria.dobre@umfcd.ro (M.-Z.D.); daciana.stefan@umfcd.ro (D.C.A.D.-S.); 2 Central Military Emergency University Hospital “Dr. Carol Davila”, 88 Mircea Vulcanescu Street, 010825 Bucharest, Romania; ioana-cristina.doicin@rez.umfcd.ro; 3 Department of Physiology, Faculty of Dentistry, Carol Davila University of Medicine and Pharmacy, 050474 Bucharest, Romania; iulia.stanescu@umfcd.ro

**Keywords:** myokines, insulin resistance, chronic inflammation, sarcopenia, thyroid dysfunction, metabolic disorders

## Abstract

Insulin resistance develops when skeletal muscle (SM), adipose tissue (AT), and the liver fail to respond adequately to insulin, a dysfunction closely intertwined with chronic low-grade inflammation. This combination leads to compensatory hyperinsulinemia, dysglycemia, and metabolic stress, driving major disorders such as type 2 diabetes, metabolic syndrome, metabolic dysfunction-associated steatotic liver disease (MASLD), and cardiovascular disease. Both adipokines and myokines are central modulators of this metabolic–inflammatory axis. In obesity, diabetes, MASLD, and thyroid dysfunction, alterations in myokines such as myostatin, irisin, fibroblast growth factor 21 (FGF-21), apelin, brain-derived neurotrophic factor (BDNF), interleukin-6 (IL-6), and interleukin-15 (IL-15) influence glucose uptake, lipid oxidation, mitochondrial function, and systemic inflammation. Exercise-induced myokines exert insulin-sensitizing and anti-inflammatory effects, whereas myostatin and tumor necrosis factor-alpha (TNF-α) promote metabolic impairment. These pathways reveal extensive crosstalk between SM and key metabolic organs—including the liver, pancreas, AT, intestine, heart, and thyroid gland. In metabolic disease, inflammation-driven changes in deiodinase activity and triiodothyronine (T3) availability further link muscle dysfunction with thyroid imbalance. The aim of this narrative review was to elucidate the complex interplay between myokines, adipokines, inflammation, and insulin resistance, and to clarify their clinical relevance in metabolic and thyroid disorders. Given this integrative role of SM, sarcopenia should be recognized as a clinical marker of metabolic or thyroid dysregulation, and preserving muscle mass through structured physical activity should be a core therapeutic target.

## 1. Introduction

Early research has revealed that insulin resistance is strongly associated with inflammation, as different studies demonstrated that pro-inflammatory mediators such as interleukin-6 (IL-6) and tumor necrosis factor-α (TNF-α) were found to have increased levels in conditions associated with insulin resistance [1,2]. Inflammation has been considered to be the body’s response to the presence of a pathogen agent or the result of tissue injury. However, recent research shows that inflammation development is not always related to these stimuli but rather influenced by different mediators that also take part in numerous physiological events, including adipokines and myokines [3].

Adipokines or adipocytokines are proteins released by the white AT that can regulate metabolic functions via endocrine, autocrine or paracrine mechanisms and can impair insulin sensitivity in target tissues [4]. Among the numerous adipokines, leptin and adiponectin are produced only by AT cells; other adipokines—such as plasminogen activator inhibitor-1 (PAI-1), monocyte chemoattractant protein-1 (MCP-1), IL-6, TNF-α and several chemokines—can also be secreted by immune or stromal cells within adipose depots, reflecting the immunometabolic complexity of adipose tissue [5].

Myokines are peptide molecules produced by specialized multinucleated cells called fibers, part of the, the largest organ in the non-obese body [6,7]. SM requires high ATP levels to sustain physical activity and therefore is the main site of glucose uptake, which is needed to support energy production [8].

Myokines have been found to contribute to muscle autocrine metabolism regulation, as well as being key players in maintaining homeostasis by enhancing insulin sensitivity, therefore having beneficial effects on glucose metabolism. Additionally, they promote glucose oxidation and have important physiological roles in myogenic differentiation and muscle mass preservation, while also contributing to the upregulation of lipid and energy metabolisms [9,10]. The endocrine roles of myokines have led to the acknowledgement of SM as a paracrine and endocrine organ [11,12]. On the other hand, myokines have an impact on muscular physiology, and they can also enable information exchange with other organs characterized due to the presence of myokine receptors, such as the liver, the heart, and bones, or with other types of tissues (e.g., AT or intestinal tissues) [6,9].

Proteomic research of the secretome in SM has determined over 100 myokines out of the 3000 identified [9,13] which are produced by muscles in response to exercise, the most studied being myostatin, decorin, irisin [7], fibroblast growth factor 21 (FGF-21), secreted protein acidic and rich in cysteine (SPARC), apelin, decorin, 3-amino-2-methylpropanoic acid (BAIBA), brain-derived neurotrophic factor (BDNF), myostatin [9], resistin, TNF-α and interleukins-6 (IL-6), -8 (IL-8), and -15 (IL-15)—the cytokines traditionally known as a pro-inflammatory mediators, but that can also be derived from myocytes in physiological conditions [14,15].

Myostatin or growth and differentiation factor-8 (GDF-8) is a member of the transforming growth factor beta superfamily, and it inhibits SM growth by decreasing protein synthesis via inhibition of the protein kinase B/mammalian target of rapamycin (AKT/mTOR) and adenosine monophosphate-activated protein kinase (AMPK) signaling pathways [16,17]. The inactivation of these pathways disrupts GLUT-4 translocation to the cell surface, as previously mentioned, which results in decreased glucose uptake [18]. Thus, myostatin can be associated with insulin resistance (IR), the connection being confirmed by previous research that revealed increased myostatin levels in obesity and enhanced insulin sensitivity and glucose uptake after myostatin expression loss [19]. Moreover, it stimulates muscle loss, being linked to sarcopenia, heart failure, and myopathy [16]. Furthermore, it can stimulate the expression of TNF-α [17], IL-1β, and IL-17 [20], hence promoting inflammation.

**Figure 1:**
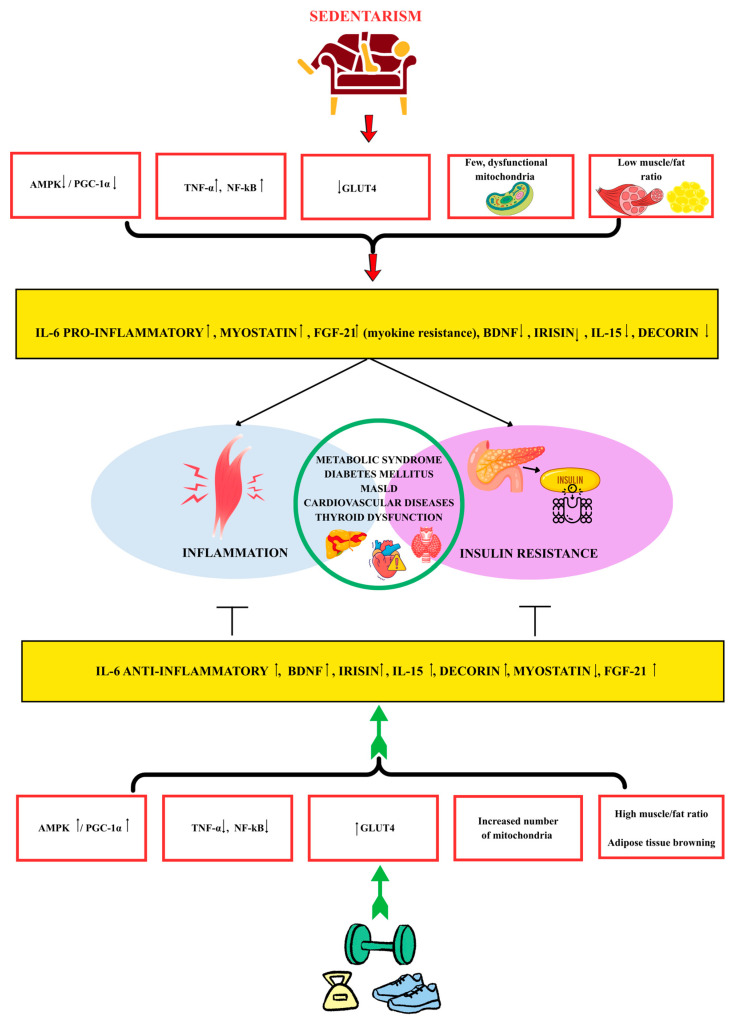
Sedentarism and physical exercise differentially shape myokine profiles, inflammation, and insulin resistance. The upward arrow (↑) indicates an increase, and the downward arrow (↓) indicates a decrease (created using Canva with a Pro license, accessed on 19 November 2025).

Figure 1 summarizes how sedentarism and physical activity differentially modulate myokine profiles, inflammation, and insulin resistance. Physical inactivity is associated with AMPK and peroxisome proliferator-activated receptor gamma coactivator 1-alpha (PGC-1α) suppression, upregulation of TNF-α and nuclear factor kappa-light-chain-enhancer of activated B cells (NF-κB), GLUT-4 inactivation, the presence of few and dysfunctional mitochondria, and a decreased muscle/fat ratio. All these alterations lead to an increased serum level of myostatin, FGF-21 and pro-inflammatory IL-6. At the same time, BDNF, irisin, IL-15 and decorin levels drop. These variations promote inflammation and insulin resistance, creating the optimal environment for the development of metabolic syndrome, type 2 diabetes mellitus (T2D), metabolic dysfunction-associated steatotic liver disease (MASLD), and cardiovascular and thyroid disorders. On the other hand, physical exercise has the opposite impact, inhibiting the development of insulin resistance and inflammation, thus having a protective effect against the aforementioned pathologies.

Irisin, a peptide consisting of 112 amino acids, is produced both by SM and AT [21], can induce the transformation of white adipose tissue into brown adipose tissue [22] and promotes GLUT-4 expression [14]. Also, a previous study showed that irisin stimulates AMPK phosphorylation, thus facilitating glucose uptake in SM [23]. Additionally, it has been found that irisin reduces gluconeogenesis [24] and can improve fatty acid oxidation via AMPK [25]. Furthermore, irisin was shown to have an anti-inflammatory role by inhibiting the Toll-like receptor 4/myeloid differentiation factor 88-mediated nuclear factor kappa-B (TLR4/MyD88-mediated NF-κB) and NLR family pyrin domain containing 3 (NLRP3) inflammasome signaling pathways in AT cells, beta pancreatic cells and macrophages, among others. Additionally, it prevents obesity by stimulating lipolysis [21].

Apelin, an endogenous peptide that acts as a ligand for the apelin receptor, also called APJ, a member of G protein-coupled receptor family [26], is a myokine that can also be considered an adipokine [27] and is also present in the brain, heart, lungs, and vascular endothelial cells. It can decrease blood pressure, exhibit anti-inflammatory properties, promote angiogenesis and tissue repair, and mitigate oxidative stress [28]. Additionally, apelin potentiated the effects of insulin by augmenting glucose transport and uptake in SM and AT [29].

MCP-1, a 76-amino-acid peptide, is an adipo-myokine considered part of the chemokine superfamily. It chemotactically attracts leukocytes, especially macrophages (the mature form of the monocytes) and B lymphocytes, traditionally having a pro-inflammatory role [30,31] that is linked to insulin resistance development [32].

BDNF is a neurotrophin involved in neuronal growth and survival. It can also be released by the satellite cells of SM, acting as a myokine that can stimulate tissue repair after injury [33]. In mice, suppression of BDNF was associated with necrosis, leukocyte infiltration, pyroptosis and hyperproduction of IL-1β, -18, -23, leading to myositis, hence suggesting the physiological protective role of this myokine [34]. Moreover, BDNF is considered to be a metabokine due to its positive effects on energy, lipid and glucose metabolism, as it increases insulin sensitivity in muscles, but also in AT and the liver [35].

Resistin has emerged as a key adipokine linking innate immunity to insulin resistance. In humans, resistin is mainly produced by macrophages infiltrating adipose tissue, and higher circulating levels have been consistently reported in obesity, type 2 diabetes and metabolically unhealthy phenotypes [36]. Large cross-sectional cohorts demonstrate that resistin correlates positively with HOMA-IR, triglyceride-rich lipoproteins and inflammatory markers, whereas adiponectin exhibits the opposite pattern, emphasizing their divergent contributions to cardiometabolic risk [37,38]. Higher resistin levels are associated with reduced muscle mass and poorer physical performance in older adults, suggesting that resistin contributes not only to hepatic and adipose insulin resistance but also to sarcopenia and inflammation-driven metabolic impairment, based on observational human evidence [39,40].

Beyond these classical mediators, several additional adipokines—including chemerin, visfatin, retinol-binding protein-4 and omentin—have gained attention for their strong metabolic and inflammatory effects. Recent human studies show that chemerin is elevated in obesity, MASLD and type 2 diabetes and correlates positively with BMI, hepatic fat and HOMA-IR in cross-sectional clinical studies [41,42].

Visfatin, predominantly secreted by visceral adipose tissue, exhibits insulin-mimetic activity but is paradoxically increased in metabolic syndrome, diabetic microangiopathy and insulin-resistant states, reflecting both compensatory and pathogenic mechanisms as indicated in clinical observational studies and mechanistic evidence [43,44,45]. These adipokines act together with leptin and adiponectin to shape the inflammatory and metabolic profile of adipose tissue and influence systemic insulin sensitivity.

The aim of this narrative review is to clearly describe and integrate current evidence on the interactions between myokines, adipokines, inflammation, and insulin resistance, and to explain their clinical relevance in endocrine–metabolic and thyroid disorders. This review focuses on the bidirectional crosstalk between inflammation and insulin resistance and the central modulatory role of skeletal muscle–derived myokines within this network. In addition, the manuscript aims to translate these pathophysiological insights into clinical practice by emphasizing sarcopenia as an early and practical indicator of metabolic and thyroid dysfunction, thereby providing clinicians with a useful framework for improved diagnosis, risk stratification, and multidisciplinary patient management.

## 2. Myokine Dysregulation in Metabolic Disease

Disruptions in myokine signaling represent one of the earliest and most informative molecular fingerprints of metabolic disease. As insulin resistance develops, skeletal muscle shifts from an oxidative, stress-resilient tissue toward an inflamed, metabolically inflexible state, altering both the quality and quantity of myokines it releases. These changes do not occur in isolation; they intersect with adipose tissue inflammation, mitochondrial dysfunction and impaired inter-organ communication, creating a biochemical environment that reinforces metabolic deterioration. Understanding how these signals become distorted provides essential insight into the mechanisms that drive insulin-resistant conditions.

### 2.1. Myokine Remodeling in Type 2 Diabetes

Type 2 diabetes (T2D) represents a prototypical chronic metabolic disorder in which persistent low-grade inflammation profoundly distorts the myokine environment and accelerates the development of insulin resistance [46]. Prolonged exposure of skeletal muscle to inflammatory cytokines induces a transition from an oxidative, AMPK/PGC-1α–driven phenotype toward a stress-responsive state dominated by NF-κB and JNK activation, mitochondrial dysfunction, and impaired metabolic flexibility [47,48]. These abnormalities disrupt glucose disposal, lipid oxidation and insulin signaling at multiple regulatory levels.

Myostatin (GDF-8), frequently elevated in obesity and early T2D, exerts potent anti-anabolic and anti-oxidative effects and signals through the SMAD (mothers against decapentaplegic homolog) family of transcription factors, particularly SMAD2/3 [49,50]. Through canonical SMAD2/3 activation, myostatin suppresses Akt phosphorylation, reduces PGC-1α activity, impairs mitochondrial biogenesis, and contributes to diminished oxidative capacity and muscle atrophy [51]. Recent clinical evidence obtained from observational clinical cohorts studies further shows that higher circulating myostatin levels correlate with lower insulin sensitivity in overweight and obese adults, supporting its role as an early biomarker of sarcopenic–metabolic dysfunction [50].

Conversely, insulin-sensitizing myokines—including irisin, IL-15 and apelin—are typically reduced in T2D. Meta-analytic data of human clinical studies show significantly lower irisin levels in T2D, consistent with impaired mitochondrial remodeling and reduced beige-fat recruitment [52]. Small-sample exercise intervention trials revealed that IL-15, a contraction-induced myokine linked to oxidative metabolism, rises acutely after exercise in humans [53] and correlates inversely with adiposity indices [54]; although T2D-specific data remain sparse, reduced IL-15 signaling is believed to contribute to diminished muscle metabolic efficiency [9]. Apelin, which improves glucose uptake and endothelial insulin action, is likewise suppressed in T2D [55,56].

These disturbances reinforce a self-sustaining inflammatory loop: NF-κB/JNK activation increases IRS-1 serine phosphorylation and blocks canonical insulin signaling [47], while mitochondrial dysfunction elevates ROS production, which further amplifies cytokine output and tissue inflammation [57]. Human proteomic analyses and early mechanistic evidence displayed the additional contribution of extracellular vesicles (EVs) from adipose tissue and skeletal to endocrine signaling. Moreover, proteomic studies in obese and diabetic individuals reveal extensive EV remodeling and impaired insulin-sensitizing cargo [58].

### 2.2. Clinical Implications and Therapeutic Modulation of Organokines

Clinically, combined myokine–adipokine profiling is increasingly explored as a tool to capture early insulin resistance and to stratify metabolic risk. Patterns characterized by elevated leptin and resistin together with reduced adiponectin associate with adverse atherogenic lipid profiles, higher remnant cholesterol and incident cardiometabolic events in large population-based cohorts [59]. These biomarker signatures are also dynamically modulated by contemporary therapies. High-level evidence obtained from meta-analyses of RCTs indicates that GLP-1 receptor agonists significantly increase circulating adiponectin and reduce leptin and resistin concentrations, in parallel with improvements in body weight, glycemic control and markers of vascular risk [60,61]. More recent clinical imaging studies and small interventional trials offered data indicating that GLP-1 receptor agonists reduce epicardial and visceral adipose tissue, improve systemic inflammatory tone and downregulate several pro-inflammatory adipokines. Emerging evidence also suggests potential benefits on muscle metabolic pathways, with promising indications that these effects may help improve muscle function, insulin sensitivity, and overall metabolic health, although data from translational studies on direct myokine remodeling are still in the early stages of investigation [62,63]. Structured aerobic or resistance training reliably enhances GLUT4 expression and insulin-stimulated glucose uptake in skeletal muscle. In addition, several recent human and translational studies, mainly small randomized or non-randomized exercise trials, have reported increases in specific myokines such as apelin and IL-15 following training, which may contribute to a more favorable myokine milieu. However, data remain inconsistent for other myokines, such as irisin, and the evidence that exercise induces a stable anti-inflammatory myokine profile remains preliminary and is supported by heterogeneous clinical findings [64,65]. Integrating these therapeutic effects with adipokine and myokine measurements may refine risk stratification and help personalize lifestyle and pharmacological interventions in metabolic and thyroid disorders.

### 2.3. Myokine Imbalance in Obesity and Sarcopenic Obesity

Obesity exemplifies a chronic inflammatory milieu in which skeletal muscle becomes both a recipient and amplifier of systemic metabolic stress [66]. Expansion of adipose depots increases secretion of IL-6, TNF-α, MCP-1, and pro-inflammatory EVs, which propagate dysfunction across metabolic tissues and re-shape the myokine output of skeletal muscle, as supported by experimental studies and human observational evidence [67]. Human data synthesized by Orioli and Thissen indicate that obesity and type 2 diabetes are characterized by an altered muscle secretome, with increased release of pro-inflammatory myokines (IL-6, IL-8, IL-15, TNF-α, MCP-1) and growth regulators such as myostatin and follistatin, changes that parallel chronic activation of inflammatory pathways and impaired oxidative/AMPK–PGC-1α signaling in skeletal muscle, as demonstrated by systematic human clinical evidence [68].

Chronic exposure of muscle to lipotoxic intermediates and inflammatory cytokines enhances IRS-1 serine phosphorylation, suppresses PI3K/Akt signaling, and impairs GLUT4 translocation, as demonstrated in mechanistic and translational studies [69,70]. Muscle-biopsy evidence in individuals with obesity shows elevated myostatin and activated SMAD signaling, leading to reduced PGC-1α, defective mitochondrial biogenesis, and intramyofiber lipid accumulation [49,71]. Reduced irisin limits thermogenic browning [72], while diminished IL-15 weakens muscle–adipose communication; human exercise-intervention studies confirm IL-15 rises with contraction and associates with oxidative phenotype markers [53,73,74].

Metabolic deterioration becomes even more pronounced in sarcopenic obesity, in which excess adiposity coexists with reduced muscle mass and strength. Low muscle quantity limits the production of beneficial myokines such as irisin and IL-15 while elevating antagonistic mediators such as myostatin [17]. Mitochondrial dysfunction is more severe in this phenotype, with reduced oxidative enzyme content, ROS accumulation, and impaired mitophagy and quality control mechanisms, as supported by experimental and translational studies [75]. Age-related pro-inflammatory senescence-associated secretory phenotype (SASP) factors exacerbate catabolism by increasing IL-6, TNF-α and proteolytic enzymes [76]. Recent observational clinical data show that obesity-associated sarcopenia is accompanied by lower irisin and higher IL-6 levels, both of which correlate with reduced handgrip strength and muscle mass [77].

Signaling impairments in sarcopenic obesity include marked AMPK/PGC-1α suppression, chronic NF-κB/JNK and SMAD2/3 activation, and disrupted autophagy–mitophagy cycles—alterations that synergistically promote IR and metabolic inflexibility [78]. Integrating muscle mass assessment with circulating myokine profiling (myostatin, irisin, IL-15) has been proposed as a strategy to identify metabolically vulnerable obesity phenotypes, supported by emerging multi-center clinical evidence [79].

Therapeutically, interventions that modulate adipokines, hepatokines and myokines and activate AMPK—such as exercise-induced organokine signaling—are increasingly recognized as approaches to mitigate adipose inflammation and improve systemic insulin sensitivity (supported by exercise-intervention trials) [21]. Incretin-based therapies further enhance metabolic outcomes: GLP-1 receptor agonists induce substantial weight loss with favorable body composition changes, while dual incretin agonists modulate adipose tissue metabolism, inflammatory tone, and adipose–muscle crosstalk, as demonstrated by high-level evidence from randomized controlled trials [80,81].

## 3. Myokine Dysregulation in MASLD

### 3.1. Sarcopenia and MASLD: Interlinked Risks and Mechanisms

Metabolic dysfunction-associated steatosis liver disease (MASLD), formerly called non-alcoholic fatty liver disease (NAFLD), is highly prevalent worldwide, impacting more than 30% of people globally [82].

Sarcopenia in cirrhosis arises from a complex interplay of metabolic, hormonal, and inflammatory factors. Contributing factors include complications of portal hypertension, physical inactivity, increased hepatic gluconeogenesis, and impaired insulin and IGF-1 signaling, elevated levels of pro-inflammatory cytokines, hyperammonemia, the use of loop diuretics, and hypotestosteronemia. Alcohol-related liver disease further exacerbates muscle wasting [83].

**Figure 2:**
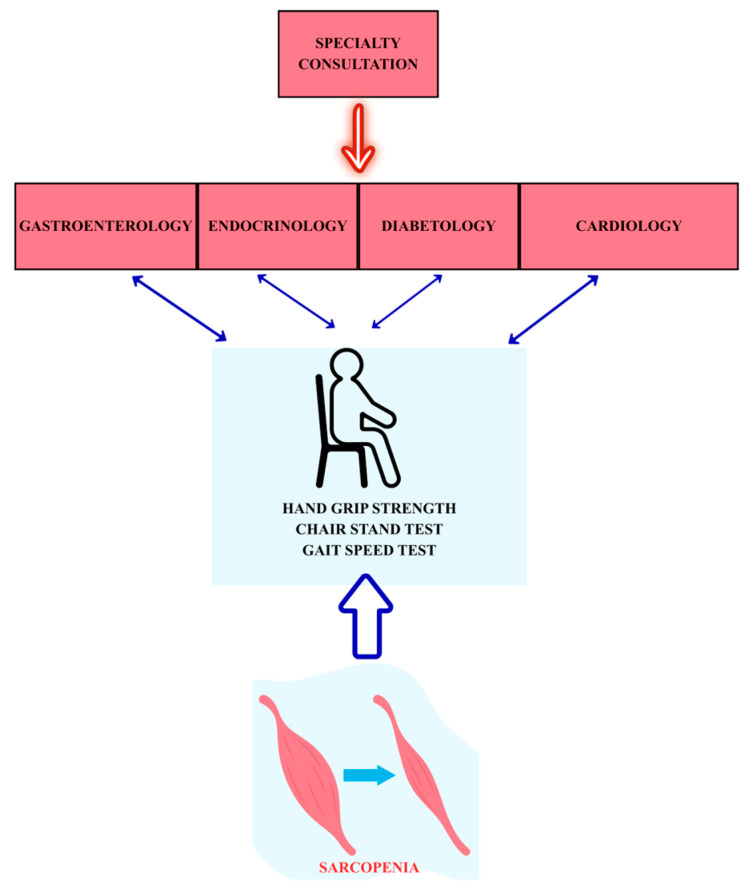
The dual relationship of sarcopenia with various metabolic and endocrine diseases (created using Canva with a Pro license, accessed on 19 November 2025).

The dual relationship between MASLD and sarcopenia, which is presented in Figure 2, is supported by numerous systematic reviews and meta-analyses of clinical studies, some of which are highlighted below. Figure 2 emphasizes the importance of incorporating routine sarcopenia screening into gastroenterology, endocrinology, diabetology, and cardiology consultations. Well-established and easily performed clinical tests, such as handgrip strength, the chair stand test, and the gait speed test, allow clinicians—including those managing metabolic and endocrine disorders—to identify sarcopenia, which can significantly impact the prognosis of metabolic diseases.

MASLD is caused by fat accumulation in the liver, leading to inflammation and possibly liver cancer. MASLD comprises different conditions, including steatosis, MASH and cirrhosis, and is linked to obesity and insulin resistance, which promote sarcopenia—the loss of muscle mass and strength [84]. Multiple meta-analyses have evaluated MASLD risk in individuals with sarcopenia and consistently shown that those with sarcopenia have a greater likelihood of developing MASLD [85,86,87,88].

Also, sarcopenia is a key factor influencing the severity of MASLD. A thorough evaluation of muscle mass, strength, and function is crucial for early detection and timely intervention in this high-risk group. When MASLD and sarcopenia occur together, they can intensify disease burden and increase the likelihood of serious complications such as falls, fractures, cardiovascular events, and higher overall mortality [89].

Skeletal muscle alterations (SMAs) and MASLD are driven by common mechanisms such as chronic inflammation, insulin resistance, and low physical activity. In MASLD, SMA worsens insulin sensitivity, contributes to greater hepatic fat buildup, and may accelerate the progression to fibrosis or cirrhosis [88,90].

It has been demonstrated that irisin acts as a protective factor and a potential biomarker for obesity-related comorbidities, while alterations in myonectin and myostatin further contribute to cardiometabolic risk in MASLD patients [91,92]. Shen et al. in a systematic review and meta-analysis, suggest that irisin may serve as a diagnostic marker and reflect disease progression [93]. Similarly, Polyzos S.A., in a study based on liver biopsy analysis in NAFLD patients, reports that elevated irisin levels may indicate a more aggressive liver disease phenotype with increased fibrogenesis and tissue damage [94].

Also, in a small cross-sectional clinical study published in 2025, it was shown that circulating levels of irisin, osteocalcin, and FGF-21 were elevated in obese individuals compared with those of normal-weight controls. Their interconnected actions suggest that these molecules may contribute to the development and progression of obesity and its associated metabolic disorders such as MASLD [95].

### 3.2. Myokines and Liver Dysfunction

Myokines are cytokines released by contracting skeletal muscle that act as key regulators of inter-organ communication and whole-body metabolic homeostasis. Bucarey et al. published a review providing an in-depth analysis of key myokines and their potential impact on liver function, highlighting multiple myokines—including myostatin, brain-derived neurotrophic factor (BDNF), fibroblast growth factor 21 (FGF-21), and irisin—that may influence hepatic physiology [96]. Also, in another review published recently, the authors highlight the myokine–liver crosstalk. They emphasize that in MASLD, Kupffer cells shift toward a pro-inflammatory M1 phenotype, while quiescent hepatic stellate cells become activated into myofibroblasts that drive fibrosis. Most myokines modulate these cell types, exerting anti-inflammatory and anti-fibrotic effects [97]. Moreover, the Muscle–Gut–Liver axis has been proposed as a critical concept, as the prevention of liver disease onset and progression requires considering the interconnected network of these organs [98].

Due to its antioxidant properties, irisin is considered a hepatoprotective peptide. It regulates crucial cellular processes such as ferroptosis, inflammasome activation, autophagy, mitochondrial fission and fusion, ER stress, and cell death [96]. Irisin promotes the browning of white adipose tissue and enhancer thermogenesis by binding to its receptor and activating downstream signaling pathways, including the AMP-activated protein kinase (AMPK) and p38 MAPK [99].

In observational studies, circulating irisin, primarily derived from skeletal muscle with a potential contribution from hepatic stellate cells depending on disease activity, is generally lower in MASLD patients compared to healthy individuals [97,100,101]. As previously noted, the rise in irisin in chronic liver disease likely represents a mechanistic damage response process, consistent with its overexpression in hepatic stellate cells in significant fibrosis; however, in advanced disease with sarcopenia, irisin levels no longer correlate with muscle mass or disease severity [101,102]. Also, an association between high irisin levels and the presence or exacerbation of cardiometabolic disorders has been shown [103].

Studies in mice have shown that FGF-21 serves as a protective myokine in MASLD, enhancing the antioxidant response and providing protection against ferroptosis through mechanisms that activate the nuclear factor erythroid 2–related factor 2 (NRF2)/heme oxygenase-1 (HO-1)/glutathione peroxidase 4 (GPX4) pathway [104,105]. Moreover, FGF-21 reduces hepatic lipid influx and accumulation through both hormonal and local signaling, preventing Kupffer cell activation and decreasing the number of lipid- and scar-associated macrophages, thereby limiting fibrogenesis [106]. Another myokine with antagonistic effects to FGF-21 is myostatin. In a translational study, elevated myostatin promoted muscle loss by enhancing protein breakdown and autophagy, linking liver dysfunction to sarcopenia [107].

Cross-sectional human studies have shown that BDNF is measurable in plasma and may serve as a biomarker for neurological disorders. While BDNF levels are reduced in obesity and type 2 diabetes, they are elevated in NAFLD and increase with disease severity [108]. Low plasma myostatin is associated with reduced pro-inflammatory cytokine expression and improved insulin sensitivity. However, elevated myostatin shows inconsistent associations: some studies link it to muscle loss, hyperammonemia, reduced protein synthesis, and lower liver stiffness [92], whereas others report that higher serum myostatin predicts poorer prognosis in liver cirrhosis by promoting collagen synthesis [109]. In cirrhosis, myostatin levels are decreased in patients with acute decompensation and acute-on-chronic liver failure (ACLF), and low serum myostatin independently predicts ACLF development and mortality, irrespective of liver disease severity or sex. A serum myostatin level below 1.280 pg/mL best predicts ACLF [110]. In contrast, an increase in myonectin may represent a compensatory response to elevated blood lipids and metabolic dysfunction. Its associations with uric acid and hypertension support its potential role as a marker of metabolic disorders and the body’s adaptive responses. Accordingly, MASLD patients with detectable serum myonectin had higher systolic blood pressure and elevated uric acid compared with those with undetectable levels [92].

In vitro studies have shown that BDNF exerts beneficial effects on the liver by promoting β-oxidation of free fatty acids and inhibiting gluconeogenesis through AMPK activation in mouse hepatocytes [111], whereas in cirrhosis, hyperammonemia has been demonstrated to activate NF-κB in skeletal muscle, leading to increased myostatin expression [107].

### 3.3. Myokines, Exercise, and MASLD

Regular moderate-to-vigorous exercise, particularly ≥5 times per week, significantly reduces the risk of developing new fatty liver and markedly increases the likelihood of resolving existing fatty liver disease [112]. According to the American College of Sports Medicine (ACSM) recommendations for MASLD, individuals should engage in ≥150 min per week of moderate-intensity physical activity (40–60% heart-rate reserve) or ≥75 min per week of vigorous-intensity activity (60–85% heart-rate reserve). Heart-rate reserve (HR reserve) is defined as the difference between an individual’s maximum heart rate and resting heart rate [113].

Experimental studies in mouse MASLD models have shown that exercise provides significant benefits in mitigating disease progression, largely by enhancing both the release and gene expression of myokines. Exercise-induced increases in irisin and FGF-21 are associated with reduced inflammation, suggesting their protective effects in MASLD [96,114,115]. Furthermore, exercise stimulates skeletal muscle secretion of interleukin-15, which improves metabolic dysfunction–associated steatosis by reducing liver macrophage and CD8^+^ T cell accumulation [116]. Myostatin expression has also been shown to decrease after acute and chronic endurance and resistance exercises in rodents [117]. Overall, exercise benefits the liver by reducing fat accumulation, improving metabolic efficiency, and supporting hepatic function. It enhances energy expenditure, promotes fatty acid oxidation, and facilitates healthier lipid droplet remodeling, reducing droplet size and increasing their contact surface with mitochondria [118].

In MASLD patients, moderate- and high-intensity exercise increases circulating myonectin levels, suggesting enhanced lipid metabolism with regular training [119]. Exercise also reduces myostatin, a key inhibitor of muscle growth, while increasing follistatin and irisin, both of which promote protein synthesis and muscle hypertrophy [120].

Both moderate-intensity continuous training (MICT) and high-intensity interval training (HIIT) provide comparable benefits in MASLD, although aerobic and resistance exercises act via distinct mechanisms. Aerobic training improves insulin sensitivity and increases energy expenditure, helping to reduce hepatic fat, whereas resistance training enhances metabolic flexibility through AMPK activation, muscle-fiber adaptations, and muscle–liver crosstalk, supporting better metabolic control. Long-term success depends on consistent adherence and integration into an individualized lifestyle plan [121].

As previously mentioned, low-volume HIIT, despite requiring much less time, produces metabolic and muscular adaptations nearly identical to those achieved with MICT in adults with obesity. However, improvements in insulin sensitivity are short-lived and disappear within a few days without exercise, indicating that the primary insulin-related benefit derives from each recent workout rather than long-term training adaptations [122].

In a comparative study conducted by Japanese researchers, the exercise group—although they lost less weight than the diet group—showed greater improvements in liver enzymes, liver fat accumulation, liver stiffness, non-esterified fatty acids, and insulin resistance. These superior benefits were linked to exercise-induced changes in myokines and adipokines, including adiponectin, myostatin, and leptin [123].

Surprisingly, recent evidence indicates that physical activity may also confer significant benefits in patients with cirrhosis [124].

The following table presents key myokines whose plasma levels are influenced by physical exercise and which modulate liver metabolism, many of them having implications in MASLD (Table 1).

## 4. Myokines and Thyroid Dysfunction

### 4.1. Role of Thyroid Hormones in Metabolic Regulation and Inflammation

Thyroid hormones (THs) are essential regulators of skeletal muscle development, metabolism, and repair. Acting through specific transporters (MCT8, MCT10), receptors (THRα, THRβ), and local activation by deiodinases (DIO2, DIO3), they modulate gene expression involved in contractile protein synthesis, mitochondrial biogenesis, and energy metabolism. Through these coordinated mechanisms, THs ensure proper muscle function, adaptation, and regeneration [141,142].

Thyroid hormones (THs) are central regulators of energy metabolism and tissue homeostasis. They act through both classical genomic and alternative nongenomic pathways to coordinate energy production, oxidative balance, and inflammatory control. During aging or cardiometabolic stress, chronic low-grade inflammation disrupts peripheral conversion of thyroxine (T4) to triiodothyronine (T3), leading to reduced T3 availability and insulin resistance. This dysfunction triggers compensatory activation of the hypothalamic–pituitary–thyroid (HPT) axis, resulting in elevated thyroid-stimulating hormone (TSH) levels [143]. With persistent metabolic stress, tissue sensitivity to THs declines, further reducing T3 bioavailability and predisposing to non-thyroidal illness syndrome (NTIS). Elevated TSHs may signal insulin resistance and metabolic syndrome, whereas NTIS is associated with catabolic states, frailty, and poor prognosis [143]. In this context, IL-37 lowers inflammation and cellular stress, helping protect thyroid function and support a more stable hormonal balance [143].

In thyroid disorders, shifts in IL-6, IL-17, IL-37, and Th17/Treg balance contribute to chronic inflammation, oxidative stress, and tissue remodeling. These immune-metabolic changes affect thyroid hormone production and signaling, linking inflammation, metabolic dysfunction, and thyroid disease progression [144,145].

Also, dysregulated adipokines such as leptin, resistin, and TNF-α promote chronic low-grade inflammation and may potentiate autoimmune responses that impair thyroid function. In contrast, exercise-induced myokines—including IL-6 and BDNF—exert anti-inflammatory, insulin-sensitizing, and immunomodulatory effects that support both thyroid and metabolic homeostasis [146].

### 4.2. Role of Thyroid Hormones in Muscle Thermogenesis: Evidence from Animal Studies

Triiodothyronine (T3) regulates key physiological processes including metabolism, thermogenesis, and tissue development. Forty years ago, a study in rats demonstrated that brown adipose tissue (BAT) contributes to systemic T3 production through DIO2-mediated conversion of thyroxine (T4) to T3, a process activated by norepinephrine [147]. In turn, T3 promotes thermogenesis by inducing uncoupling protein 1 (UCP1) expression via thyroid hormone receptor β1 (TRβ1) signaling. Prolonged T3 exposure enhances BAT expansion and thermogenic capacity through THRA-mediated proliferation of adipocyte [147].

In mice, it was shown that in skeletal muscle, triiodothyronine (T3) promotes oxygen consumption by stimulating mitochondrial activity and myogenic gene expression [148]. Moreover, T3 also influences muscle fiber composition, driving the conversion of slow-twitch (type I) to fast-twitch (type II) fibers, thereby enhancing both glycolytic and oxidative capacity. Mechanistically, T3 induces microRNA miR-133a1, which represses TEAD1, a transcription factor maintaining slow-twitch fiber identity [149].

### 4.3. Clinical Evidence for the Bidirectional Relationship Between Myokines and the Thyroid–Endocrine System

Skeletal muscle functions as an endocrine organ, releasing myokines that regulate muscle metabolism and repair. These myokines also influence whole-body physiology by controlling body weight, reducing low-grade inflammation, improving insulin sensitivity and inhibiting tumor growth [150].

Alterations in thyroid status profoundly affect muscle structure and function. Hypothyroidism is associated with weakness, stiffness, oxidative stress, and impaired glycogen metabolism, resulting in a slow, painful myopathy [9,151]. In contrast, hyperthyroidism increases protein turnover and mitochondrial activity, leading to muscle atrophy, weakness, and reduced exercise capacity—changes that are typically reversible upon restoration of euthyroidism. Elevated free T4 levels in hyperthyroid patients have been linked to a higher risk of sarcopenia [152].

Patients with subclinical hypothyroidism show milder alterations in this continuum—characterized by elevated TSHs with normal T4 and T3—alongside increased systemic inflammation, oxidative stress, impaired glucose regulation, and lipid abnormalities [153]. While often asymptomatic, some individuals may experience subtle hypothyroid symptoms such as muscle weakness, cramps, stiffness, or fatigue [154].

In more severe or chronic conditions (e.g., cardiovascular or renal disease), NTIS develops, marked by low T3 and elevated reverse T3 (rT3) without a compensatory rise in TSH. Studies on postmortem samples from intensive care patients revealed reduced hepatic deiodinase type 1 (D1) and increased deiodinase type 3 (D3) activity in the liver and skeletal muscle, particularly under poor tissue perfusion. These enzyme alterations diminish local T3 production while accelerating its degradation, resulting in intracellular T3 deficiency. This deficit impairs muscle metabolism and contributes to functional decline typical of NTIS [155].

### 4.4. Myokines, Adipokines, Insulin Resistance and Metabolic Regulation in Thyroid Dysfunction

Both adipokines (secreted by adipose tissue) and myokines (released by skeletal muscle) act as inflammatory and metabolic mediators, including interleukin IL-6, IL-8, and monocyte chemoattractant protein-1 (MCP-1), which regulate immune and energy balance [7].

As shown by cellular-level investigations, skeletal muscle also influences insulin secretion by releasing myokines that act on pancreatic β-cells. For example, IL-6 indirectly increases glucagon-like peptide-1 (GLP-1) expression in intestinal L cells, boosting insulin release [156]. In diabetes, this adaptive response is impaired, promoting insulin resistance and metabolic dysfunction through p38 MAPK and PI3K/PKC signaling pathways) [15].

Both hypo- and hyperthyroidism contribute to insulin resistance and impaired glucose metabolism, effects that can occur even in subclinical or mild hormonal imbalances. Elevated free T3 may enhance glucose uptake but also promotes hepatic glucose production and lipolysis, worsening insulin sensitivity. With aging, free T3 and T4 levels decline; subclinical hypothyroidism appears less harmful in older adults, while subclinical hyperthyroidism increases fracture and mortality risk. Slightly higher T3 levels may help maintain glucose balance, though factors such as adiposity, cortisol, comorbidities, and medications more strongly affect insulin sensitivity in this population [157].

It is known that adipokines interact dynamically rather than acting in isolation. Based on clinical data, elevated adiponectin inhibits the secretion of TNF-α and IL-6, whereas TNF-α suppresses adiponectin and enhances IL-6 production. Strenuous exercise reduces leptin levels, with diabetic patients showing better responses, and increases adiponectin, particularly in overweight individuals. Physical activity also stimulates muscle-derived IL-6 release, proportional to muscle mass and exercise intensity and decreases TNF-α levels and reduces insulin resistance risk [158].

Thyroid dysfunction further disrupts muscle metabolism. It was demonstrated that in hypothyroid myopathy, low T3 and T4 impair mitochondrial function, glycogen utilization, and oxidative metabolism, leading to insulin resistance, fast-twitch fiber atrophy, and slowed contraction. Accumulation of glycosaminoglycans and connective tissue contributes to stiffness and hypertrophy, while oxidative damage and low carnitine cause weakness and fatigue [151]. Both hypo- and hyperthyroidism are associated with insulin resistance and dysglycemia, even within reference hormone ranges. Elevated T3 may transiently enhance glucose uptake but also increases hepatic glucose production, intestinal absorption, glycogenolysis, and lipolysis, ultimately worsening insulin sensitivity [9].

### 4.5. Exercise, Myokines and Metabolic Regulation in Thyroid Dysfunction

Review studies, including trials, clinical interventional, and observational studies, have summarized evidence that physical activity influences systemic metabolism through myokine secretion. During muscle contraction, myokines—such as myonectin, irisin, BDNF, and decorin—are released, exerting anti-inflammatory effects on visceral adipose tissue, enhancing mitochondrial function, and improving insulin sensitivity. In contrast, physical inactivity reduces myokine release and increases the risk of metabolic disorders. [146,159]. Exercise training in hypothyroidism has received limited research attention compared with other chronic conditions. In women with hypothyroidism receiving levothyroxine therapy, aerobic training, resistance training, and combined aerobic–resistance training all improved thyroxine concentrations, lipid profile, and physical aspects of quality of life. However, the combined aerobic–resistance program produced the greatest reductions in thyroid-stimulating hormone and the most pronounced improvements in mental quality of life, whereas aerobic training alone elicited the largest gains in exercise capacity [160].

In a small-sample clinical intervention study, it was demonstrated that recreational exercise in patients with Hashimoto’s thyroiditis modulates inflammatory and growth-related proteins linked to muscle health. It reduces fibroblast growth factor 5 (FGF-5), enhances interleukin-15 receptor alpha (IL-15R α) and hepatocyte growth factor (HGF) expression, and downregulates tumor necrosis factor-like weak inducer of apoptosis (TWEAK), supporting muscle metabolism, regeneration, and protection from wasting. Exercise also modifies inflammatory profiles across disease severity groups, with the most favorable effects seen in levothyroxine-treated patients, suggesting synergy between hormone therapy and physical activity [161].

Regular physical activity lowers pro-inflammatory cytokines such as tumor necrosis factor alpha (TNF-α), IL-6, and C-reactive protein (CRP). Its effect on thyroid hormones varies with intensity, but moderate, supervised exercise improves lipid metabolism, mood, sleep, and physical performance, particularly in subclinical hypothyroidism. In hyperthyroidism, exercise tolerance is reduced but improves after restoring euthyroidism [162].

During exercise, transient IL-6 elevation induces IL-1 receptor antagonist (IL-1ra) and IL-10, inhibiting pro-inflammatory cytokine signaling and creating an anti-inflammatory environment [145,163]. Hyper- and hypothyroid patients tolerate different exercise programs, and physical activity itself can modify thyroid hormone levels, with aerobic and anaerobic training recommended differently for each condition [164].

Exercise also improves the adipokine profile, increasing adiponectin—which enhances energy expenditure and insulin sensitivity—and reducing leptin, resistin, TNF-α, and IL-6 levels [165]. Aerobic and resistance training lower oxidative stress and improve glucose metabolism, while muscle-derived IL-6 release during contraction enhances insulin sensitivity and reduces the risk of insulin resistance. Elevated adiponectin suppresses TNF-α and IL-6, while TNF-α inhibits adiponectin production, reflecting a dynamic adipose–muscle signaling network [158,165].

### 4.6. Interrelationship Between Type 2 Diabetes, Thyroid Dysfunction

The relationship between diabetes mellitus and thyroid dysfunction is supported by numerous observational studies. Several of these are highlighted below, focusing on the role of myokines as the link between these disorders.

IL-6, the best-known representative of the cytokine superfamily of molecules, is renowned for its pro-inflammatory actions. However, this interleukin can also be produced by the adipose tissue or by SM (in response to physical exercise), acting as an adipo-myokine and exerting anti-inflammatory roles. The pleiotropic functions of IL-6 include myoblast proliferation, SM growth, and muscle repair after damage. Furthermore, it acts as a muscle energy sensor [166]. IL-6 was found to increase insulin sensitivity by stimulating glucose uptake and to stimulate lipolysis and oxidation of free fatty acids. Nevertheless, in obesity-associated conditions the pro-inflammatory activity of this cytokine can lead to insulin resistance [14].

Among myokines, interleukin-6 (IL-6) remains a central integrator of metabolic and inflammatory cues. During acute exercise, IL-6 functions as a myokine that activates AMPK, enhances glucose uptake, and promotes fatty acid oxidation [167]. In contrast, chronic low-grade IL-6 elevation in T2D engages the JAK/STAT3–SOCS3 pathway, driving SOCS3 overexpression, IRS-1 inhibition and downstream PI3K/AKT suppression [52,168,169]. Translational human muscle-biopsy studies consistently confirm the link between sustained IL-6 signaling and impaired muscle insulin action [170,171].

Similarly to IL-6, TNF-α is a synonym of inflammation and has even been considered the link between insulin resistance and obesity [172]. However, unlike IL-6, TNF-α can only increase insulin resistance by inhibiting AMPK signaling pathway and decreasing glucose uptake, impairing insulin signaling and lipid metabolism [173].

Chronic inflammation and oxidative stress, common in diabetes, impair deiodinase activity, reduce T4-to-T3 conversion, and promote DNA damage and tumor progression through NADPH oxidase–mediated reactive oxygen species generation. The resulting biochemical pattern—elevated free T4 with normal or low free T3—resembles non-thyroidal illness syndrome (NTIS), reflecting impaired peripheral hormone activation [174]. Severe insulin resistance has been associated with an eightfold higher prevalence of papillary thyroid carcinoma compared with the general population, suggesting a role in cancer initiation, although tumor aggressiveness appears unrelated to insulin resistance severity [175]. Chronic low-grade inflammation in type 2 diabetes activates MAPK signaling and increases cytokines such as TNF-α, IL-6, and IL-8, which enhance thyroid cell proliferation, angiogenesis, and survival, favoring tumor development [176]. Hyperinsulinemia and insulin resistance further promote thyroid carcinogenesis by activating the insulin/IGF-1–MAPK–PI3K signaling pathways, stimulating cell proliferation and inhibiting apoptosis [177]. Additional contributors, including elevated TSH, chronic hyperglycemia, oxidative stress, and vitamin D deficiency, accelerate tumor progression. Obesity amplifies these processes through adipokine imbalance, with high leptin and low adiponectin enhancing inflammation and proliferation, thereby increasing thyroid cancer risk in diabetic individuals. In patients with type 2 diabetes, thyroid dysfunction is associated with elevated circulating levels of visfatin, chemerin, resistin, and inflammatory markers, with milder changes observed in subclinical disease [178].

Oxidative stress, reflected by increased malondialdehyde, is an independent risk factor for hypothyroidism and contributes to thyroid cell damage. Higher free T3 concentrations in insulin-resistant patients may represent a compensatory mechanism to counteract metabolic stress and reflect disease severity [179]. Impaired oxidative capacity promotes lipid spillover into muscle, intensifying adipose inflammation and elevating systemic IL-6/TNF-α, which further deteriorate muscle insulin sensitivity [180]. Multiple observational clinical studies report inverse associations between circulating myostatin and insulin sensitivity, handgrip strength, and muscle function [50,181,182]. Meta-analyses and pooled human clinical studies also link low irisin and IL-15 levels with poorer glucose tolerance, lower mitochondrial enzyme activity, and higher cardiometabolic risk. [52,53,183].

Additional large-scale evidence reinforces these patterns. Elevated IL-6 and TNF-α consistently associate with reduced insulin sensitivity and visceral adiposity in large population-based studies [184]. Higher circulating myostatin independently predicts lower insulin sensitivity in adults with overweight or obesity [181], while reduced irisin levels accompany impaired glucose tolerance and lower aerobic capacity [52]. Collectively, this dysregulated cytokine–myokine profile appears to reflect—and potentially contribute to—the muscle–adipose communication defects characteristic of metabolic disease.

Many researchers consider interleukin-6 (IL-6) a marker of metabolic syndrome rather than a direct causal factor; however, during exercise, muscle fibers release IL-6 independently of tumor necrosis factor-α (TNF-α). Additionally, exercise elevates insulin-like growth factor-1 (IGF-1), an essential mediator of muscle protein synthesis that is often reduced in individuals with sarcopenia. Collectively, these myokines contribute to improved muscle mass and strength [120].

In young patients with type 1 diabetes, cytokine profiles are largely comparable to those of healthy individuals, except for elevated interleukin (IL)-4, tumor necrosis factor alpha (TNF-α), IL-18, vascular endothelial growth factor (VEGF), and angiogenin, alongside decreased IL-10 levels. In the subgroup with coexisting autoimmune thyroiditis, angiogenin was further increased, distinguishing this cohort and suggesting a shared pro-angiogenic and inflammatory mechanism linking diabetes and thyroid autoimmunity [185].

A cross-sectional study reported that systemic inflammation increases with visceral obesity and insulin resistance, with monocyte chemoattractant protein-1 (MCP-1) levels correlating with both the homeostatic model assessment of insulin resistance (HOMA-IR) and antithyroperoxidase antibodies (ATPO), indicating a potential link between metabolic inflammation and autoimmune thyroiditis [179]. Additionally, an epidemiological study showed that type 2 diabetes is associated with a higher prevalence of thyroid disorders—particularly malignant nodules—with risk increasing with age, female sex, and underlying autoimmunity. Disruption of the hypothalamic–pituitary–thyroid axis in diabetes further affects insulin sensitivity and glucose regulation, whereby hyperthyroidism exacerbates hyperglycemia, while hypothyroidism increases the risk of hypoglycemia [176].

Together, these findings highlight the reciprocal interactions between thyroid hormones, skeletal muscle, and adipose tissue, with physical activity acting as a key modulator of inflammation, energy metabolism, and insulin sensitivity. Both thyroid hormone excess and deficiency disrupt muscle homeostasis through distinct metabolic and molecular pathways, underscoring the essential role of thyroid hormones in preserving muscle integrity. Overall, the coexistence of type 2 diabetes and thyroid dysfunction exemplifies a complex metabolic, hormonal, and inflammatory interplay. The overlap with non-thyroidal illness syndrome and the higher prevalence of thyroid abnormalities in diabetic patients highlight the importance of systematic thyroid screening and integrated metabolic–endocrine assessment.

## 5. Myokines in Muscle Regeneration and Atrophy

Satellite myogenic cells are muscle stem cells formed in the intrauterine life and located in the basal lamina. They are responsible for the repair of skeletal muscle after damage. Under normal conditions, satellite cells are undifferentiated and in quiescent state, but specific stimuli trigger their activation in physiological or pathological conditions, leading to division and progenitor formation, proliferation, and differentiation [186]. Subsequently, they fuse forming myotubes that resemble the original muscle fibers. Muscle regeneration is regulated by the *Pax* genes [187]. The most important role in satellite cells activation is played by myokines [188].

In hyperthyroidism, as well as in T2D, muscle weakness and atrophy are frequent challenges [189,190]. Hindered maturation of myofibers together with fibrosis impair muscle regeneration in diabetes, making foot ulcers a common complication. Tissue remodeling is promoted by inflammation mediators and is initiated by the activation of the complement system which leads to leukocytes infiltration. The increased mobilization of neutrophils in the inflammatory region stimulates IL-1β, IL-6, TNF-α, and IFN-γ release, which in turn stimulate macrophage and mast cell chemotaxis, as well as the proliferation and differentiation of satellite cells [189].

TNF can stimulate myogenesis via activation of p38 and exert anti-inflammatory roles [125]. During muscle regeneration, the cooperation between TNF-α and interferon γ (IFN-γ) results in the conversion of the pro-inflammatory state of the macrophages to an anti-inflammatory one [120].

In KO mice, BDNF was found to regulate the differentiation of satellite cells and therefore myogenesis [191]. Irisin and myostatin exert opposing effects on muscle [138]. Myostatin stimulates muscle atrophy and several drugs targeting it have been developed, but muscle strength was not improved [191]. Myostatin promotes muscle degradation by inhibiting satellite cell activation and muscle regeneration, contributing to sarcopenia [138]. Myostatin produces cell-cycle arrest by blocking the transition from phase G1 to S via cyclin-dependent kinase 2 upregulation, hence inhibiting satellite cells proliferation and differentiation [189]. In contrast, irisin supports muscle growth through ERK signaling and counteracts myostatin’s catabolic and pro-inflammatory actions, including AMPK-mediated anti-inflammatory pathways [138]. Irisin was found to activate satellite cells and synthesis of proteins [191]. Reduced irisin levels therefore enhance myostatin-driven muscle loss, whereas higher irisin levels help protect against sarcopenia [138].

## 6. Challenges in Interpreting Circulating Myokine Levels

Several challenges arise for researchers and clinicians when assessing circulating myokine levels, including the complex reciprocal and antagonistic interactions among myokines, the high cost of ELISA kits, and the possibility of myokine resistance, which may obscure the interpretation of serum concentrations.

Myokine resistance refers to a state in which circulating myokine concentrations are elevated, yet target tissues fail to elicit the expected biological response. FGF21 and irisin exemplify this phenomenon in obesity, where their serum levels are often increased while their metabolic actions—such as enhancing glucose homeostasis and promoting lipid oxidation—are attenuated. This pattern suggests reduced tissue sensitivity to these hormones. For FGF21, downregulation of the co-receptor β-Klotho and FGF receptors in adipose tissue and liver impairs formation of the functional receptor complex, resulting in weakened ERK1/2 signaling [192]. In the case of irisin, elevated circulating levels may reflect a compensatory response to diminished tissue responsiveness, although augmented secretion from expanded adipose and muscle depots in obesity remains a plausible alternative [193].

Myokine research in critically ill patients shows that physical rehabilitation has important systemic effects. Although interest has increased, studies are limited by small sample sizes, inconsistent therapy protocols, and uncertainty about which myokines and time points to measure [194].

Combining regular physical exercise with strategies that improve insulin sensitivity may offer effective, anti-inflammatory treatments to improve metabolic health and clinical outcomes [195].

Future myokine research will focus on defining precise molecular mechanisms to personalize exercise prescriptions and develop exercise-mimicking drugs. Myokines are expected to become key biomarkers for tailoring training intensity and monitoring therapeutic responses in chronic and neurological diseases. Further studies are needed to clarify their interactions with the nervous system and other organ-derived “kines” (e.g., hepatokines, batokines) to optimize integrated, multi-organ health interventions [126].

Consequently, lifestyle modification and a reduction in sedentary behavior should be considered fundamental components of patient management. Muscle-derived regulatory RNAs, metabolites, and engineered myokines will offer emerging therapeutic avenues for chronic metabolic and endocrine disorders.

## 7. Conclusions

Skeletal muscle plays a central role in metabolic and endocrine health, and increased muscle mass provides substantial protective effects in patients with metabolic and thyroid disorders. In the management of metabolic diseases, weight control through dietary interventions should be complemented by strategies to increase muscle mass, such as structured physical training. These benefits arise not only from improved metabolic capacity but also from the secretion of exercise-induced myokines, which contribute to the regulation of inflammation, insulin resistance, oxidative stress, and muscle regeneration, all of which are key pathogenic mechanisms in these conditions. The close bidirectional interactions between skeletal muscle and major metabolic organs—including the liver, thyroid gland, adipose tissue, and pancreas—highlight the importance of maintaining muscle mass and function. Significant gaps remain in understanding the early responses of myokines and the impaired release of these signaling molecules during critical illness. Future research should aim to standardize interventions and measurement timing, identify sensitive biomarkers, support the development of myokine-targeted drugs, formulate personalized exercise prescriptions, and enable multi-center clinical validation to enhance patient outcomes. In particular, elucidating the interactions between exercise-induced myokines, especially irisin, and adipokines in the regulation of metabolism, inflammation, and muscle regeneration will be essential. Defining reliable biomarkers and key signaling pathways will be crucial for the development of targeted therapeutic strategies for obesity, diabetes, and muscle atrophy. Given this integrative role of skeletal muscle, clinicians across all specialties should recognize sarcopenia as an important indicator of potential metabolic or thyroid dysfunction and address it proactively in clinical practice.

## Figures and Tables

**Table 1:** Exercise-regulated myokines affecting hepatic metabolism in MASLD.

Myokine	Physiological Effects Related to Hepatic Metabolism	Effect of Exercise on Myokine Plasma Level	Myokine Plasma Level in MASLD	Reference
IL-6	Protects against chronic diseases associated with low-grade inflammation	Increased IL-6 with anti-inflammatory effect	Increased IL-6 with pro-inflammatory effect	[125]
	Increases lipolysis and sensitivity to insulin in fatty tissue			[126]
	Increases glycogenolysis and lipolysis in the liver			[127]
	Optimizes the production of insulin in the pancreas			[128]
Myostatin	Favors sarcopenia Increases collagen synthesis	Decreased	Increased	[117] [129]
Irisin	Stimulates fat burning	Increased	Decreased	[99]
	Improves insulin sensitivity			[96]
	Inhibits fibrogenesis (suppression of hepatic stellate cell activation)			[130]
IGF-1	Promotes muscle growth and regeneration	Increased	Decreased	[131]
IL-15	Maintains muscle mass, improves glucose and lipid metabolism	Increased	Decreased	[116]
Apelin	Enhances hepatic glucose uptake via AMPK activation; improves hepatic insulin sensitivity; reduces steatosis by promoting fatty acid oxidation and limiting lipotoxicity	Increased (particularly in response to high-intensity interval training and endurance exercise).	Decreased in MASLD and obesity; lower levels correlate with insulin resistance, hepatic steatosis and endothelial dysfunction	[132,133,134,135]
FGF-21	Insulin sensitization	Increased	Increased (myokine resistance)	[106]
	Diminished immune cell infiltration			[106]
	Increased fatty acid oxidation			[129]
BDNF	Contributes to feeding and energy metabolism	Increased	Decreased	[136]
	Healthy aging and longevity			[137]
	Reduces inflammation by cholinergic anti-inflammatory pathway			[138]
	Decreases blood glucose levels			[139]
Decorin	Anti-inflammatory and anti-fibrotic effects	Increased	Decreased	[140]

## Data Availability

No new data were created or analyzed in this study. Data sharing is not applicable to this article.

## Abbreviations

The following abbreviations are used in this manuscript:

ACLF	acute-on-chronic liver failure
AKT	protein kinase B
AMPK	AMP-activated protein kinase
APJ	apelin receptor
AT	adipose tissue
ATP	adenosine triphosphate
ATPO	antithyroperoxidase antibodies
BAIBA	3-amino-2-methylpropanoic acid (β-aminoisobutyric acid)
BAT	brown adipose tissue
BDNF	brain-derived neurotrophic factor
CD8	cluster of differentiation 8
CRP	C-reactive protein
D1	type 1 deiodinase
D3	type 3 deiodinase
DIO2	type 2 iodothyronine deiodinase
DIO3	type 3 iodothyronine deiodinase
DNA	deoxyribonucleic acid
ER	endoplasmic reticulum
EVs	extracellular vesicles
FGF-5	fibroblast growth factor 5
FGF-21/FGF21	fibroblast growth factor 21
GDF-8	growth and differentiation factor 8 (myostatin)
GLP-1	glucagon-like peptide 1
GLUT-4/GLUT4	glucose transporter type 4
GPX4	glutathione peroxidase 4
HGF	hepatocyte growth factor
HIF-1	hypoxia-inducible factor 1
HO-1	heme oxygenase 1
HOMA-IR	homeostatic model assessment of insulin resistance
HPT	hypothalamic–pituitary–thyroid axis
IFN-γ	interferon gamma
IGF-1	insulin-like growth factor 1
IGF-IR	insulin-like growth factor 1 receptor
IL-1β	interleukin-1 beta
IL-1ra	interleukin-1 receptor antagonist
IL-4	interleukin-4
IL-6	interleukin-6
IL-8	interleukin-8
IL-10	interleukin-10
IL-15	interleukin-15
IL-15R α	interleukin-15 receptor alpha
IL-17	interleukin-17
IL-18	interleukin-18
IL-23	interleukin-23
IL-37	interleukin-37
IR	insulin resistance
IRS	insulin receptor substrate
IRS-1	insulin receptor substrate 1
JAK	Janus kinase
JAK/STAT3	Janus kinase/signal transducer and activator of transcription 3 pathway
JNK	c-Jun N-terminal kinase
MASLD	metabolic dysfunction-associated steatotic liver disease
MASH	metabolic dysfunction-associated steatohepatitis
MAPK	mitogen-activated protein kinase
MCP-1	monocyte chemoattractant protein-1
MCT8	monocarboxylate transporter 8
MCT10	monocarboxylate transporter 10
miR-133a1	microRNA-133a1
M1	classically activated (pro-inflammatory) macrophage phenotype
NADPH	nicotinamide adenine dinucleotide phosphate (reduced form)
NAFLD	nonalcoholic fatty liver disease
NF-κB	nuclear factor kappa-light-chain-enhancer of activated B cells
NLRP3	NLR family pyrin domain containing 3
NRF2	nuclear factor erythroid 2–related factor 2
NTIS	non-thyroidal illness syndrome
PAI-1	plasminogen activator inhibitor 1
PGC-1α	peroxisome proliferator-activated receptor gamma coactivator 1-alpha
PI3K	phosphatidylinositol 3-kinase
PKC	protein kinase C
RNA	ribonucleic acid
ROS	reactive oxygen species
SASP	senescence-associated secretory phenotype
SGLT2	sodium–glucose cotransporter 2
SM	skeletal muscle
SMA	skeletal muscle alterations
SMAD	SMAD family of signal transducer proteins
SMAD2/3	SMAD family member 2/3
SOCS3	suppressor of cytokine signaling 3
SPARC	secreted protein acidic and rich in cysteine
T2D	type 2 diabetes
T3	triiodothyronine
T4	thyroxine
Th17	T-helper 17 cell
THs	thyroid hormones
THRα	thyroid hormone receptor alpha
THRβ	thyroid hormone receptor beta
TLR4	Toll-like receptor 4
TNF-α	tumor necrosis factor alpha
Treg	regulatory T cell
TRβ1	thyroid hormone receptor beta 1
TSH	thyroid-stimulating hormone
TWEAK	TNF-like weak inducer of apoptosis
UCP1	uncoupling protein 1
VEGF	vascular endothelial growth factor
